# A scoping review of student Athletes’ perspectives on dual career policies, provisions and challenges

**DOI:** 10.3389/fspor.2025.1566208

**Published:** 2025-06-03

**Authors:** Marianna De Maio, Simone Montenegro, Olga Papale, Sofia Serafini, Iris Prestanti, Pascal Izzicupo, Beatrice-Aurelia Abalasei, Tara Alonso del Hierro, Burak Çalışkan, Angela Di Baldassarre, Håkon Ege, Antonio José Figueiredo, Barbara Ghinassi, Higinio González-García, Ionut Onose, Raluca-Mihaela Onose, Matteo Perissinotto, Andrea Molinari, Amaia Ramírez-Muñoz, Antonio Sánchez-Pato, Nemanja Stanković, Nenad Stojiljković, Ilvis Abelkalns, Mojca Doupona, Laura Capranica, Andrea Fusco

**Affiliations:** ^1^ Department of Human Sciences, Society and Health, University of Cassino and Lazio Meridionale, Cassino, Italy; ^2^ Department of Medicine and Aging Sciences, University “G. d’Annunzio” of Chieti-Pescara, Chieti, Italy; ^3^ Faculty of Physical Education and Sports, Department of Physical Education, “Alexandru Ioan Cuza” University of Iasi, Iasi, Romania; ^4^ Faculty of Health Sciences, Sport Sciences Department, International University of La Rioja (UNIR), Logroño, Spain; ^5^ Collective Innovation AS, Oslo, Norway; ^6^ Research Unit for Sport and Physical Activity, Faculty of Sport Sciences and Physical Education, University of Coimbra, Coimbra, Portugal; ^7^ European Athlete as Student (EAS) Network, Ghaxaq, Malta; ^8^ Department of Innovative Technologies in Medicine and Dentistry, University “G. d’Annunzio” of Chieti-Pescara, Chieti, Italy; ^9^ TECNODEF Research Group, Faculty of Education, Department of Physical Education and Health, Universidad Internacional de La Rioja (UNIR), Logroño, Spain; ^10^ OKKAM srl, Trento, Italy; ^11^ NÌKE Research Group. Faculty of Health Sience, Universidad Internacional de La Rioja (UNIR), Logroño, Spain; ^12^ Vice-Rectorate for Research, NÌKE Research Group, Faculty of Health Sience, Universidad Internacional de La Rioja (UNIR), Logroño, Spain; ^13^ Faculty of Sport and Physical Education, University of Niš, Niš, Serbia; ^14^ Department of Teacher Education, University of Latvia, Riga, Latvia; ^15^ Faculty of Sports, Department of Sport Sociology and History, University of Ljubljana, Ljubljana, Slovenia; ^16^ Department of Movement, Human and Health Sciences, University of Rome “Foro Italico”, Rome, Italy

**Keywords:** dual career, student-athletes, education, dual career services, support

## Abstract

Dual career (DC) athletes face significant challenges in balancing dual demands of academic and athletic commitments. A scoping review of 25 studies published between 2014 and 2024 included data from over 3,000 student-athletes across 23 countries, with 88.5% focused on European contexts. Most adopted qualitative (52%) or quantitative (44%) approaches, with one study (4%) using a mixed method. Findings, synthesized using PRISMA guidelines, addressed logistical, social, financial, tutorship, curricula, and policy aspects. Recurring barriers included a lack of flexible educational programs, insufficient financial aid, and limited access to proximate sports and facilities. Social support systems, such as mentorship and institutional committees, emerged as essential for engagement and reducing isolation. European athletes frequently cited the need for improved financial support, highlighting scholarships and fee waivers. During the COVID-19 pandemic, e-learning strategies supported educational adherence and reduced stress, emphasizing their potential as flexible tool for addressing DC demands. However, disparities in policy implementation and service provision persist, with studies identifying cohesive institutional strategies for DC athletes. These findings underscore the need to develop harmonized frameworks across Europe, prioritizing integrated logistical planning, expanded financial support and tailored curricula. Broader perspectives from stakeholders are needed to enable DC athletes to thrive academically and athletically.

## 1 Introduction

Sport has undergone rapid social and economic growth, playing a crucial role in promoting personal development and individual fulfilment, especially among youth. Furthermore, sport participation offers individuals opportunities for physical, mental, and emotional growth, while ensuring and preserving educational and vocational rights, as well as opportunities to succeed in society and integrate into the labour market after the end of their sports career (1). In this context, a growing interest has emerged in the promotion of combining education and sport commitments (2).

Recently, the term “dual career” (DC) was introduced to describe the specific challenges athletes face in combining sport and study activities (3). Achieving elite status in sport is a complex task requiring years of intensive training sessions and competitions (4). Indeed, athletic careers can require a long-term practice from 20 to 30 h per week in training and competition activities, combined with 30 or more hours per week in educational demands (5). The dual demands placed on DC athletes often result in psychological (6, 7) and physical stress (8), as well as time constraints that make it difficult to excel in both sport and educational activities, potentially leading to burnout and reduced engagement over time (9, 10). DC athletes may encounter significant difficulties when attempting to combine their sportive activities and educational demands, leading to a high risk of sport or academic dropouts (7, 11–13). Thus, to prevent the athletes' integration from being compromised by their sporting careers, it is necessary to address athletes' rights and opinions regarding their access to supports in education and sport. For this reason, and to achieve the right of DC athletes to balance their sporting and educational demands, the European Union (EU) (14) has promoted several strategies at local, regional, national, and transnational levels, highlighting logistic, social, financial, tutorship, curricula requirements, and policy items.

Following this line, scientific research has focused on several DC aspects, with systematic, scoping, narrative, and critical literature reviews summarizing findings and highlighting future challenges on psychological and social aspects of DC athletes, high school student-athletes' mental health, barriers and routines of DC paths, mentoring DC athletes, efforts of parenting DC athletes, DC gaps in women's sport, and transnational collaborations of DC European funded projects (3, 6, 7, 15–22). Among the several DC challenges (17, 19, 23–26), educational institutions are encouraged to provide DC services that meet a minimum level of quality, including the integration of DC strategies into the institutions' overall vision, mission, and policies (12, 14, 27). To promote the dual career of university athletes-students, the EU-funded More Than Gold project provided guidelines for higher education institutes regarding financial support, infrastructure, mentors/tutors, curricula requirements, social support, and other support such as peer-to-peer assistance, DC observatory, promotion of student-athletes achievements, organization of DC events, seminars, workshops, etc. (28).

In this era of rapidly advancing technologies and a growing emphasis on accessibility and inclusion, incorporating flexible educational programmes and examinations for athletes through blended and distance learning and recognizing informal learning credits earned through participation in sports have become both essential and unique (16). Also, the presence of tutors and counsellors, key figures in the development of new learning models with an in-depth understanding of challenges and opportunities in pursuing a DC, is essential to provide personalized support for athletes (29, 30). Nevertheless, regarding the socialization aspect, DC athletes are often more limited than their peers (31) due the amount of sport and educational requirements. Logistics has also been highlighted as an essential topic. Specifically, sports centres and educational buildings, or accommodations such as sleeping facilities and cafeterias, should be located close to each other to enhance athletes' mobility (12). Moreover, it should also be noted that some DC athletes experience financial burdens related to sporting travel and university fees, which make it impossible to fully participate in both educational and athletic commitments (23). As a result, many student-athletes experience a sense of isolation and struggle to effectively balance their educational and athletic responsibilities (6, 15, 16, 18–21).

Although previous researches has highlighted the need to carry out studies on DC to gain a perspective of athletes’ the difficulties during their academic path (31), no comprehensive review on this topic has been conducted to summarize DC interventions and policy development at higher educational level. Based on the European DC guidelines for universities (14, 28, 32) the present study adopt a scoping review methodology to summarize the available evidence and to highlight potential gaps dealing with student-athletes' specific opinions on logistic, social and financial supports, assistance and tutorship, curricula requirements, and policies domains in tertiary education (33). Significant, updated, and comprehensive information could provide a clear picture of the main thematic areas related to the implementation of university DC programmes and offer suggestions to scholars for future research and to academic authorities for the implementation of key DC priorities (32).

## 2 Materials and methods

The methodology used was a scoping review that allows for the categorization and the identification of key aspects of a specific topic, highlighting gaps in the scientific literature and serving as a starting point for future researches, interventions, and policy development (34). As methodological guidance, authors followed the specific criteria described in the Preferred Reporting Items for Systematic Reviews and Meta-Analysis (PRISMA) statement (35).

### 2.1 Eligibility criteria and search strategy

To carry out the present scoping review, studies were considered eligible for inclusion if they (I) focused on DC athletes at the university level; (II) were peer-reviewed articles; (III) collected information about the thematic areas such as logistic support, assistance and tutorship, curricula requirements, social support, financial support, other supports, and policies; and (IV) were related to the COVID-19 pandemic. On the other hand, the following exclusion criteria were considered: (I) reviews; (II) conference abstracts; (III) book chapters or guideline documents; (IV) grey literature; and (V) studies not written in the English language.

The exclusion of grey literature, conference abstracts, book chapters, and guideline documents was adopted to ensure the inclusion of studies with peer-reviewed quality standards, methodological transparency, and replicability. Although such sources may provide preliminary or contextual insights, they often lack sufficient methodological detail and are not subject to rigorous peer-review processes, which could compromise the reliability and comparability of findings.

Regarding the identification of scientific articles, a computer-based search was conducted from Scopus (version December-2025), Web of Science (version January-2025) and PubMed (version January-2025) databases from April 2024 to November 2024. The 2014–2024 timeframe was selected to capture the most recent decade of research, reflecting current policies, support systems, and challenges faced by DC athletes, and to ensure the inclusion of studies potentially addressing the impact of the COVID-19 pandemic on this population.

To organize and combine terms, the Context-How-Issues-Population (CHIP) tool (36) was used. In particular, keywords and terms related to DC athletes, including both males and females, were grouped for the selection of studies' abstracts (Table 1).

**Table 1:** Keywords organized with the CHIP (context; how; issues and population) tool.

Context	Research in dual career athletes
How	Method: “qualitative” OR “quantitative” OR “mixed” OR “interviews” OR “questionnaires” AND
Issues	Dual career: “dual career” OR “dual-career” or ‘‘Elite Sport” AND Education: “college” OR “university” Themes: “support” OR “assistance” OR “tutorship” OR “curricula” OR “financial support” OR “policies” OR “facilities” OR “programmes” Period: “COVID-19” OR “pandemic” OR “coronavirus” OR “lockdown”
Population	Male and Female: “athletes” OR “sportsperson”

### 2.2 Identification and selection of relevant studies

**Figure 1:**
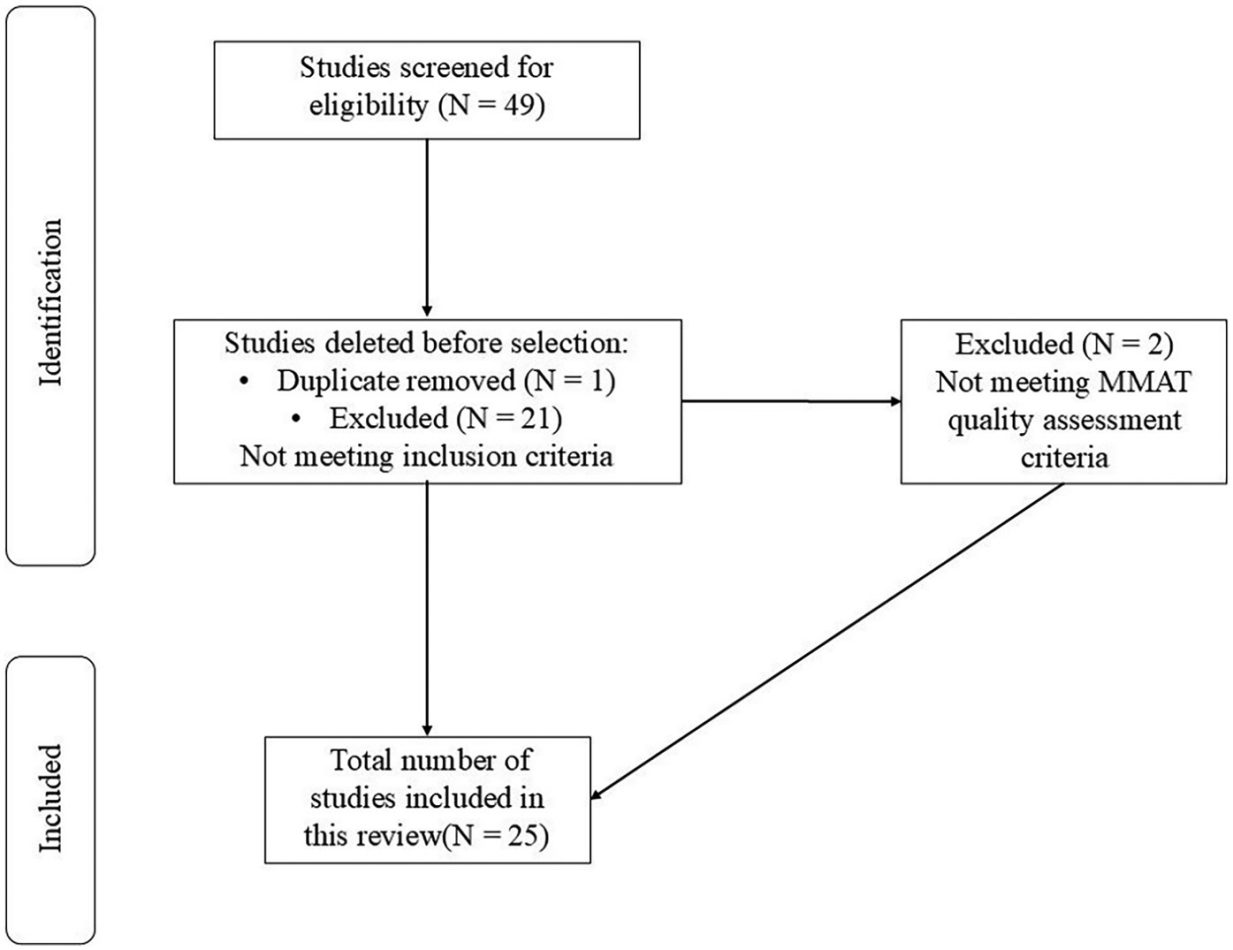
Systematic search, inclusion and exclusion of articles. MMAT, mixed methods appraisal tool.

To guarantee a systematic approach, strength, and validity of the present search methodology, the PRISMA's three steps (identification, selection, and inclusion) were assessed. Prior to the screening session, and to ensure procedural proficiency and consistency among the authors, a tutorial was organized. This session covered aspects about the thematic areas and their respective items. Finally, authors were provided with a user schematic guide. Once the potential studies were identified and their references were downloaded and collected in the bibliographic management Mendeley software (Mendeley Desktop v1.19.8-dev3), duplicates were eliminated. Subsequently, studies were screened by titles and abstracts, and full-text were analyzed to evaluate eligibility, with studies not meeting the inclusion criteria being excluded. During this phase, all authors participated, and any discrepancies were resolved by a DC expert author. Additionally, to maintain transparency in this process, a PRISMA flowchart (Figure 1) was created to document the decisions made during the screening process.

### 2.3 Coding and data extraction

To facilitate data cleaning, a data extraction tool referring to key characteristics of studies such as authorship, year of publication, participants and sport modality, study focus, design and methodology, and themes in the form of a table was developed (3) (Supplementay Table S1). The first and second authors were responsible for this data cleaning, with supervision from other authors. The included articles were arranged alphabetically by the authors' surnames.

### 2.4 Assessment of the quality of included studies

The quality of the present research methodology for the involved articles was assessed using the Mixed Methods Appraisal Tool (MMAT) (37), a critical appraisal tool designed for the evaluation stage of systematic mixed studies reviews, which include qualitative, quantitative, and mixed methods studies. The document consists of a checklist section composed of two generic screening questions and five specific ones for each type of study, with responses limited to “yes”, “no”, or “can’t tell”. A single “yes” corresponds to 20%; two “yes” to 40%; three “yes” to 60%; four “yes” to 80% and five “yes” to 100% of the MMAT criteria. Studies were also classified as very low (0%–19%), low (20%–39%), acceptable (40%–59%), moderate (60%–79%) or high (80%–100%) quality. Two independent reviewers screened each study, with an independent third-party researcher involved in case of disagreements.

The MMAT quality assessment of the involved studies by qualitative, quantitative and mixed methods is shown in Supplementary Table S2.

## 3 Results

From the electronic search, forty-nine studies were retrieved. After eliminating duplicates and excluding studies that did not meet the inclusion criteria, twenty-seven studies were identified. Moreover, following the MMAT quality assessment, two studies were removed. Finally, a total of twenty-five studies, published between 2014 and 2024, were included in the present scoping review for analysis. The main results and characteristics of interest for each study are presented in Supplementary Table S1.

The following sections provide an overview of the characteristics of DC studies, followed by a narrative synthesis of the knowledge generated through these studies.

### 3.1 Description of study characteristics

The twenty-five included studies addressing the logistic, assistance, tutorship, social and financial supports, policies, and curricula requirements for DC themes were carried out across five different continents, with 88.5% focused on European countries. Two studies were conducted during the COVID-19 pandemic (27, 38) and two studies focused on student-athletes with disabilities (39, 40). Regarding the study design, the selected works employed mainly qualitative (52%) and quantitative (44%) methodologies, and one (4%) study a mixed methodology combining semi-structured interviews and surveys (41). The most common techniques used for data collection were questionnaires/surveys and semi-structured interviews, with two studies utilizing focus groups (25, 42). With regard to the studies' focus, logistics, assistance, and tutorship supports are the most commonly assessed aspects. Lastly, based on the MMAT quality assessment, most studies were rated as high (80%–100%), three studies (38, 43, 44) as moderate (60%–79%) and one (45) as acceptable (40%–59%) quality.

### 3.2 Scope of DC studies

The section presents a narrative synthesis of the knowledge related to student-athletes' perceptions, including logistic, assistance, tutorship, social and financial supports, as well as policies and curricula requirements. A total of 25 studies mainly focused on managing DC demands. The predominant methodological approach used form 13 studies was qualitative, while 11 studies using a quantitative approach. Additionally, only 1 study adopted a mixed method approach. The studies covered and merge a wide topic of research topics, including DC student athletes and logistics (*n* = 18); assistance (*n* = 13); tutorship (*n* = 21); social (*n* = 18) and financial (*n* = 17) supports; policies (*n* = 9); and curricula requirements (*n* = 19). Therefore, managing a DC is often seen as challenging, especially when balancing the demands of both sport and educational activities (39, 45–49). Student-athletes are deeply connected to both their school and sport for several reasons, highlighting the need for enhanced support networks and services tailored to their educational responsibilities (44). DC student athletes frequently face several issues, particularly when they are pursuing both academy and sports goals. Success in both demands could be influenced by the time management (training sessions, exams, competitions and/or travel), institutional support (flexible schedule) and/or geographical mobility (accommodations and academic building). The findings of the analyzed studies revealed that the most reported barriers for DC include the absence of flexible programmes and exams (44, 50, 51), and the long distance between sport centres and educational buildings (25, 45, 52). Addressing these challenges require a cohesive and comprehensive approach including universities sport organizations and governments to provide dedicated resources in supporting DC model. On the other hand, less frequently reported are the absence of tutors or counsellors (53, 54) and social support structures such as seminars, workshops, meetings with parents and/or coaches, institutional DC committees, and peer-to-peer support (18, 25, 45, 55). The reviewed literature revealed that structured tutorship and peer-to-peer programs could provide a valid DC network, enabling athletes to excel in both demands by offering both emotional and professional support. Some analyzed studies also emphasized limited financial support (25, 31, 45). Balancing academic responsibilities with the rigorous demands of trainings and competitions require not only time and dedication but also financial resources. Many student-athletes, as well as families, could face economic difficulties, as their schedules often prevent them from taking part-time jobs to support themselves. In fact, drawing from the interview data, DC student athletes expressed their disappointment regarding the lack of comprehensive financial assistance available in supporting their dual commitments. Add to this, the lack of clear national policies (25) contributes to relevant discrepancies in their effectiveness. Evidence indicated that DC athletes encountered unharmonized policy support system among Europe. The student-athletes foresee the implementation of these aspects as essential for assisting in managing time, overcoming DC-related challenges, and preventing potential dropouts. Despite the negative impact of the COVID-19 lockdown on sports and study activities, increased e-learning opportunities represented a flexible strategy for DC (27), with student-athletes demonstrating better adherence to educational programs and perceived their DC in a positive manner (38).

## 4 Discussion

The main findings of this scoping review underscore an evolving scientific dialogue on logistic, social and financial supports, assistance and tutorship, curricula requirements, and policies domains concerning DC using different methodological approaches, substantiated by an upward trend for DC. According to Edmonson and McManus (56), the DC research could be considered entering its intermediate stage, in line with other DC topics (17). Specifically, the included studied present different methodological approaches, mainly characterized by interviews or not-validated and reliable tools to generalize findings over large-scale, cross-sport, and cross-country contexts (17). However, structured in its DC scope, the present study provides a starting point for understanding the DC support phenomenon and calls for empirical studies providing relevant information on the effectiveness of DC supports at academic level by considering both the student-athletes’ and academic DC experts' opinions across Europe (57).

In the last decade, there has been an increasing volume of publications on DC as a multi-faceted nature of the DC phenomenon, highlighting several key aspects of the student-athletes' needs in balancing sport and educational careers (2, 19, 58). Despite the EU guidelines and actions for the development of DCs and the athletes' rights to a holistic development through formal high level education, the scientific literature highlighted deficiencies in the implementation of academic support for student-athletes (2, 14). A large-scale evaluation revealed that the main barriers for DCs are logistical challenges, lack of social and financial supports, insufficient assistance and mentorship, and issues related to curriculum requirements and policies. Even though the student-athletes' opinions are essential to identify needs and anticipate solutions that create an environment supporting their DC, the opinions of DC academic experts are equally crucial to identify challenges in implementing a DC supportive environment at university level (57).

Logistic support represents a crucial aspect in DC lifestyles (25, 45, 52). It involves the organizational and practical resources provided to help athletes combining their educational and sporting requirements, for example, easily accessible sport, accommodation, and sport facilities (14). According to the European Study on Minimum Quality Requirements for DC Services (12), educational facilities such as study rooms and e-libraries, accommodation options like sleeping facilities and cafeterias, and training centres should be located within a maximum travel time of fifteen minutes to reduce stress and enhance mobility for student-athletes. Furthermore, DC relies on specific equipment, facilities, and environmental conditions, yet educational resources often fail to meet these needs (55). To fill this gap, although scientific literature have yet highlighted the importance of institutional support (19), stronger relationships between sports bodies and academic institutions should be established to make available nearby facilities to help student-athletes (59). Since student-athletes spend a significant amount of time traveling between home, educational buildings, and training venues, institutions are also encouraged to address logistics by offering or implementing specific transportation arrangements (31). In considering the globalized sport phenomenon, another important aspect involves sleeping accommodations, which could be useful for the relocation or migration of student-athletes (60). Conversely, only 18% of European student-athletes live in student accommodation (59). Therefore, prioritizing DC perspective is crucial to effectively support student-athletes in their overall development.

Social support refers to an entourage providing emotional, informational, and practical assistance to student-athletes encompassing family, peers, coaches, and institutional sources (25, 55). Specifically, students are encouraged to build positive relationships with peers to foster confidence, enhance academic performance, and create a supportive environment. Indeed, institutions have responsibilities to allocate resources for learning opportunities, student-athlete engagement programs and services that promote active involvement (55). However, the significant amount of time and effort that student-athletes dedicate to their sport can often disconnect them from their educational peer groups, resulting in a perception of limited social life outside training (44). In considering the important role of non-athlete classmates in helping student-athletes connecting with academic life and form friendships outside of their athletic domain, institutions could promote peer-to-peer tutoring, especially for first year student-athletes who need to adapt to a new context (25, 61). Moreover, consistent with searches (62), institutional DC committees could be established to facilitate dialogue among DC stakeholders, such as teachers, educational staff, coaches, and parents, while information on DC-related issues could be shared through seminars, workshops, and meetings (63). In delivering high-quality education, educational institutions might reinforce or introduce social actions to facilitate DC (14), also involving parents of student-athletes prepared to enhance the cooperation with the sports organizations and other stakeholders (21, 25, 64, 65). In this respect, it could be also useful to consider the EU-funded DC multilingual educational platform for parents of DC athletes (66).

The scientific literature also emphasizes financial support structures for student-athletes, addressing the many ways that institutions and sports organizations provide financial aid to help them balance academic and athletic commitments (23, 25, 31, 45). Student-athletes' financial support refers to resources provided to help them managing costs associated with both their educational and athletic pursuits, such as scholarships, tuition fee remission, salaries, and sponsorships (67). In this regard, many EU Member States offer sports scholarships to students, providing financial support throughout their university studies and enabling them to balance higher education with athletic pursuits (14). However, these scholarships do not cover financial assistance for certain sport-related expenses, such as equipment or travel. In fact, studies reported that high-performance athletes and their families often face long-term financial challenges, sometimes beginning at a young age (14). This aligns with previous findings that underline the need of sustained financial support over time, often as discontinuous and inadequate (68). The financial pressures associated with supporting young high-performance athletes in both sport and educational pathways could also lead to DC dropouts (69). Therefore, scholarship programs might include criteria encouraging athletes to excel educationally or athletically in exchange for rewards, including budgets for student-athletes planning to travel abroad for sports and/or education. Additionally, contributions from private organizations to these scholarships could be simulated through tax deductions.

In the context of seeking new learning models for student athletes, institutions are invited to create more inclusive, flexible, and effective educational experiences, empowering them to succeed educationally while fulfilling their athletic potential. This model includes the role of tutors, who are responsible for activities that assist and support student athletes academically (30). An effective tutorship system for DC student-athletes intellectually and emotionally motivates them to find solutions to challenges and invest the necessary effort in completing required tasks. In this context, tutors play a crucial role in positively influencing athletes' personal motivation and self-encouragement. Tutors offer guidance, share knowledge and experiences, and promote self-discovery through an approach that emphasizes low pressure (19, 50, 70). Conversely, the absence of such a figure hinders student-athletes in their learning processes, as they need proper supervision and support (53, 71). This supervision system must be adaptable to the individual needs of the student-athlete, both personally and professionally. Therefore, the tutoring system demonstrates the importance of developing innovative learning models to help athletes successfully attend educational courses (30). Moreover, when sporting activities increase, scientific literature highlights the importance of establishing an informal relationship with a tutor to mediate with academic staff and teachers in finding creative solutions, leveraging social communication channels. Through personal interactions, teachers can be more willing to offer additional work in case of missed classes, along with online consultations, support, and other tool valuable for athletes (72). For all these reasons, DC presents a challenge for educational environments, which must develop innovative ways to reorganize their knowledge and adapt learning models offered to students. In this sense, adaptable, integrated, and proactive DC programmes, based on intra- and inter-departmental cooperation and professional services, represent best practices to build a solid DC. In this respect, it could be also useful to consider the EU-funded DC toolkit for educating service providers (73).

Another key aspect of DC addressed is curricula requirements. According to the European DC recommendations (74), student-athletes require flexible curricula, personalized study plans, and distance learning options, particularly when they face conflicting schedules due to sports commitments. Since the COVID-19 pandemic, remote learning is no longer a new concept, and institutions should develop e-learning systems that include, for example, recorded lessons. These systems must provide continuous access, enabling distance teaching, educational flexibility, and ensuring future opportunities for adequate support for student-athletes (55). In fact, incorporating self-regulated remote learnings can enhance academic performance by reducing stress, helping student-athletes balance academic and athletic responsibilities, and increasing their sense of autonomy. Moreover, by awarding academic credits for sports-related activities (e.g., training, competition, and leadership roles), institutions can acknowledge the value of the skills and experiences gained through sports. This recognition reduces the workload for student-athletes, allowing them to focus on both their education and their sporting careers more effectively. Institutions might also offer credits or academic recognition for major sporting achievements, such as representing their institutions in national or international competitions. Hence, when not available, DCs may miss these vital elements (16, 75).

The European Commission (14) recommends Member States develop national DC guidelines that consider the specificities of their sport and education systems, as well as the cultural diversities within their countries. However, at present, national recommendations have been published in Sweden and in Italy (48, 76), with a few other countries regulating the principles of sport and education (e.g., Portugal 2019, 2025 and Spain 2007), yet a systematic DC-approach is often lacking at the European education level (12). Despite some institutions established policies outlining the expected behavior and responsibilities of student-athletes (e.g., maintaining academic integrity, upholding athletic standards, and adhering to institutional rules), the present scientific overview highlights that the term “policy” is still not well defined, resulting in unequal treatment of European student-athletes and unharmonized data across Member States (12, 31).

In conclusion, the scientific literature analyzed in this scoping review suggests that the student-athlete's needs in relation to logistics, social, and financial supports, as well as assistance, tutorship, curricula requirements, and policies, remain critical and require further attention. To implement effective European strategies, universities should try to overcome specific cultural and organizational regulations and establish structured cooperation for DC eligibility process, recognition, service provision, and monitoring procedures (77).

### 4.1 Practical implications

The findings of the present review substantiate the need of DC academic support to facilitate a holistic development of athletes. The definition of DC objectives and the feasibility of implementation strategies are grounded on their relevance and availability of resources. In this regard, the EU-funded More Than Gold project developed guidelines for higher educational institutions, which offer suggestions and practical directions to realise actions also in presence of limited resources (28, 32). Actually, universities are not only responsible for implementing DC policies and provisions based on the needs and expectations of their student-athletes, but also must make the athletes aware of the DC opportunities they offer (31). In this respect, universities should enable successful cooperation with sport bodies and DC stakeholders to publicize their commitment in helping athletes pursuing a higher degree, whereas scholars should engage in future research collaborations to investigate the acquisition of DC policies and provisions across tertiary education.

### 4.2 Limitations and future lines of research

This study presents several limitations. First, DC is a multi-faceted process that must be framed and managed within different contexts and cultures (70). Relevant information regarding DC cannot be fully understood by analyzing only student-athletes' views and expectations. Key actors that play a role in supporting and guiding student-athletes throughout their academic and athletic journeys include family, coaches, tutor, peers, and government bodies. In fact, integrating the opinions of key actors could facilitate the creation of a supportive system that enables student-athletes to succeed academically while pursuing their athletic goals. Second, the sample was limited to university student-athletes. The integration of multiple educational levels in future research would provide a more holistic perspective on the existing DC knowledge. Extending the scope of research would facilitate the examination of more articles and a more diverse set of data, thus enriching insights into the issues addressed in this scoping review. Consequently, it is recommended that future studies consider the limitations identified in this research as a starting point for further investigation, aiming to obtain a clearer and more exhaustive understanding of DC services and associated challenges.

## 5 Conclusions and future studies

This scoping review collected and analyzed studies focusing on student-athletes' opinions regarding logistical, social, and financial support, assistance and tutorship, curricula requirements, and policy-related domains, to obtain a global perspective of the difficulties encountered by student-athletes in developing their DC while enrolled in college. The main findings highlight the need to reinforce or introduce new actionable policies to support the status of DC student-athletes and to promote meaningful changes, especially in the aforementioned topics. Based on the evidence emerged from the present review, future research should aim to incorporate the perspectives of key stakeholders (e.g., academic tutors, coaches, parents, and institutional decision-makers) in order to foster a systemic and multidimensional approach to DC management. Moreover, the scope of investigation should be expanded by including student-athletes from various educational levels, thereby enabling a more comprehensive and longitudinal understanding of the phenomenon. It is also recommended that empirical studies assess the effectiveness of existing support measures, alongside comparative analyses of national contexts with differing levels of DC policy implementation. Finally, particular attention should be paid to the long-term impact of DC experiences on psychosocial well-being, career transitions, and professional integration, with the aim of informing the development of tailored and sustainable interventions.
