# Supplementary material for: A scoping review of student Athletes’ perspectives on dual career policies, provisions and challenges
Source: Front Sports Act Living. 2025 Jun 3;7:1566208. doi: 10.3389/fspor.2025.1566208 (PMC12170630; doi:10.3389/fspor.2025.1566208)
Supplement: Supplementary file 1 [file Table1.docx]

Supplementary Material

# Supplementary Table 1. Characteristics of the involved studies (N=25)

| Reference | Participants | Context | Type of study | Method of data collection | Theme | Key Findings |
| --- | --- | --- | --- | --- | --- | --- |
| Abelkans et al. 2019 | 14 student-athletes (8 males and 6 females) | Latvia | Qualitative | Semi-structured interviews | Logistic, assistance, tutorship, social and financial supports, policies and curricula requirements | Recommendations developed for higher education institutions |
| Abenza-Cano et al. 2020 | 150 elite SA-s (50% males and 50% females; age 25.29 ± 4.67 years) | Spain | Quantitative | ESTPORT questionnaire | Assistance and tutorship, curricula requirements, social support, other support/policies | Student-athletes provided by dual-career programs showed better adaptation compared with student-athletes from an ordinary pre-Olympic year |
| Capranica et al. 2022 | 77 athletes (35 females and 42 males; age: 20–25 years); in individual (N = 50 athletics, karate, judo, skeleton, triathlon) and team (N = 27; field hockey, hockey, soccer, rugby, volleyball) sports | Italy, Latvia, Portugal, Romania and Spain | Qualitative | Face-to-face focus groups | Logistic, assistance, tutorship, social and financial supports, policies and curricula requirements | Identified 31 key statements on athletes’ needs, assistance, and dual career policies via focus groups in 5 countries |
| Conde et al. 2023 | 109 athletes (males: 52.7% and females: 44.6%; age: 26.1 ± 4.6 years) | Spain | Quantitative | ESTPORT questionnaire | Logistic, assistance, tutorship, social and financial supports and curricula requirements | Sports tutor role highly valued, especially by national-level athletes; male athletes fell more behind in studies |
| Condello et al. 2019 | 426 athletes (males: 46%, females: 54%) | Africa, America, Asia, Europe, and Oceania | Quantitative | FISU-EAS on-line survey | Logistic, assistance, tutorship, social and financial supports and curricula requirements | Low awareness of dual career programs; parents and coaches identified as main supporters; need for a transnational network |
| Domingo et al. 2024 | 208 DC athletes, (116 females) | France, Germany, Sweden, Finland and Spain | Qualitative | Semi-structured qualitative interview  Questionnaire  Survey | Assistance, tutorship and financial supports and curricula requirements | Key benefits agreed by students and staff: free facility use, justified absences, and adaptable study pace |
| Fuchs et al. 2016 | 221 student-athletes (46.9% females and 53.1% males; age 23.4 ± 3.6 years) | Austria, Estonia, Finlandia, Italy and Slovenia | Quantitative | Questionnaire | Assistance and tutorship, curricula requirements, social support | Female athletes more academically engaged; need for academic flexibility and online learning; weak dual career policies |
| Gjaka et al. 2024 | 121 student-athletes (bachelor, masters, PhD and sports; 63.6% males and 36.4% females) | Republic of Kosovo | Quantitative | Self-administered modified version of FISU-EAS questionnaire | Logistic, assistance, tutorship, financial, social supports and curricula requirements | Poor awareness of dual career programs; main challenges: limited leisure time and academic overload |
| Hallmann et al. 2019 | 277 student-athletes (mentees; mean age: 26.2 years) | Germany | Qualitative | The Mentor Role Instrument online survey | Assistance and tutorship supports | Mentoring positively supported athletes’ transition to post-sport careers; mentors played multiple supportive roles |
| Henriksen et al. 2019 | 2 student-athletes | Denmark | Qualitative | Semi-structured interviews  Observations  Documents | Logistic, assistance, tutorship, financial, social supports and policies | Importance of coordinated, individualized environments with sport-first philosophy and athlete-centered approaches |
| Izzicupo et al.2021 | 467 student-athletes (57% males and 43% females; age: 21.6 ± 4.7 years) | Italy, Romania, Spain, Latvia, Portugual, and other | Quantitative | Survey | Logistic, assistance, tutorship, social and financial supports, policies and curricula requirements | Sport and academic commitments helped student-athletes cope with the emergency of the COVID-19 pandemic |
| Izzicupo et al. 2022 | 71 HEI experts | Croatia, Denmark, Ireland, Italy, Latvia, Poland, Portugal, Romania, Serbia, Spain, Sweden and Switzerland | Quantitative | Survey | Logistic, assistance, tutorship, social and financial supports, policies and curricula requirements | Priorities: mentorship, psychological support, flexible programs, and awareness initiatives on dual career in universities |
| Johnston et al. 2024 | 17 SAs (mentees and peer mentors; sport: hockey, soccer, football, track and field, wrestling, volleyball, basketball, and rugby) | Canada | Qualitative | Semi-structured interviews | Logistic, assistance, tutorship and social supports and curricula requirements | Peer mentoring supported first-year student-athletes’ transitions; highlighted strengths and areas for improvement |
| Kerštajn et al. 2023 | 51 Norwegian (18 females and 33 males) and 66 Slovenian student-athletes (31 females and 35 males) (sports: cross-country skiing, ski jumping and biathlon; age: 23.28 ± 4.54 years) | Norway and Slovenia | Qualitative | On-line SAMSAQ-EU questionnaire and live interviews | Assistance and tutorship supports | Student-athletes are highly motivated towards academic and sports career |
| Linnér et al. 2019 | 71 student-athletes (individual sport: athletics, cross country skiing, curling, golf, triathlon, n = 54 and team sport: floorball, ice hockey, handball, n = 17; females: 49 and males: 22; age: 25.21 ± 2.95 years) | Sweden | Qualitative | DCCQ-A  and DCCQASc questionnaires | Assistance, tutorship and financial supports and curricula requirements | Swedish student-athletes needed to improve dual career skills; perceived moderate-to-good coping with dual career challenges. |
| Maciá-Andreu et al. 2023 | 203 student-athletes with disabilities | Spain, Portugal, Italy, Ireland and Romania | Quantitative | ESTPORT, EBBS and AIMS questionnaires | Logistic, assistance, tutorship, social and financial supports, policies and curricula requirements | Perceived barriers differ by disability and professional status; athlete identity stronger in professionals |
| Mateo-Orcajada et al. 2022 | 100 pre-Olympic student-athletes (41% males and 59% females; age 24.86 ± 5.99 years) | Spain | Quantitative | ESTSPORT questionnaire | Logistic, assistance, tutorship, social and financial supports and curricula requirements | Dual career scholarship and the implementation of programs to consider sex, sport modality or type of scholarship granted showed better results obtained |
| Nikander et al. 2020 | 6 student-athletes | Finland | Qualitative | Semi-structured interviews  Observation  Documents | Logistic, assistance, tutorship, social and financial supports and curricula requirements | Changes in the organisational culture are necessary and dual career recommendations should be integrated into the environment to provide resources |
| Nyberg et al. 2023 | 274 former world-class athletes (42% females and 58% males;  age: 39.92 ± 6.25 years) | Sweden | Qualitative | Questions on DCs programme | Financial support and policies | Athletes face limited dual career assistance in reaching élite sport’s level, with financial support commonly improving only the ending of their athletic careers |
| O'Neil et al. 2020 | 248 collegiate student-athletes competing in the NCAA Division (baseball, basketball, football, golf,  soccer, softball, swimming/diving, tennis, track and field/cross country, volleyball; 157 females and 91 males; age: 17-24 years) | United States | Quantitative | SCQ-2, EDLS, ABQ, SVS, SWLS questionnaires | Logistic and social supports and curricula requirements | Dual commitment profiles linked to better well-being and engagement; need for stronger academic support networks |
| Rossi et al. 2021 | 11 athletes and 200 elite athletes | Germany | Mixed methods | Semi-structured interviews  Survey | Logistic, assistance, tutorship, social and financial supports and curricula requirements | Intrapersonal constraints most limiting; structural facilitators improved dual career outcomes; pressure had a negative effect. |
| Ryba et al. 2014 | Transnational student-athletes (3 males and 3 females; age: 25 – 36 years) | Finland, Norway and Denmark | Qualitative | Semi-structured interviews | Logistic support | Identified three transnational dual career pathways; further research needed on migrant student-athletes |
| Tekavc et al. 2015 | 12 athletes (6 males and 6 females; sports: swimming (n = 6) and  basketball (n = 6)) | Slovenia | Qualitative | Semi-structured interviews | Logistic, assistance, tutorship, social and financial supports, policies and curricula requirements | Athletic career intertwined with other life domains; academic transitions were key challenges |
| Thompson et al. 2022 | 19 student-athletes (10 males and 9 females; age: 16.5 ± 0.7 years; hockey n = 4, netball n = 4, football n = 4, rugby n = 3 and basketball n = 4). | Grait Britain | Qualitative | Focus groups with student-athletes | Logistic, assistance, tutorship, social and financial supports and curricula requirements | Sport school promoted holistic development but presented risks; essential to involve multiple stakeholders |
| Vaquero-Cristóbal et al. 2023 | 162 student-athletes with (n = 79; age: 24.34 ± 5.80 years) and without (n = 83; age: 24.07 ± 5.59 years) disabilities | Spain | Quantitative | ESTPORT questionnaire | Logistic support | Women and individual athletes reported more barriers; scholarships crucial for overcoming dual career difficulties |

DC = dual career; ABQ = Athlete Burnout Questionnaire; ESTPORT = Perceptions of dual career student-athletes; DCCQ-A = Dual Career Competency Questionnaire for Athletes; DCCQASc = Dual Career Competency Questionnaire for Athletes with scenario extension; EDLS = Engagement versus Disaffection with Learning Scale; HEI = higher education institution; NCAA = National Collegiate Athletic Association; SAMSAQ = Student Athletes’ Motivation toward Sports and Academics Questionnaire; SCQ-2 = Sport Commitment Questionnaire-2; SVS = Subjective Vitality Scale; SWLS = Satisfaction with Life Scale.
