# Supplementary material for: A scoping review of student Athletes’ perspectives on dual career policies, provisions and challenges
Source: Front Sports Act Living. 2025 Jun 3;7:1566208. doi: 10.3389/fspor.2025.1566208 (PMC12170630; doi:10.3389/fspor.2025.1566208)
Supplement: Supplementary file 2 [file Table2.docx]

Supplementary Material

# Supplementary Table 2. MMAT quality assessment profile for qualitative, quantitative and mixed studies

|  | **Screening questions** | | | | | | **Qualitative study** | | | | | | | |  | |  |
| --- | --- | --- | --- | --- | --- | --- | --- | --- | --- | --- | --- | --- | --- | --- | --- | --- | --- |
| **Reference** | S1. Are the objectives and/ or research questions clear? | | | S2. Does the data collected address the research objectives and/ or research questions? | | | 1.1. Is the qualitative approach appropriate for answering the research objective and/or research question? | 1.2. Are the qualitative data collection methods appropriate for addressing the research objective and/or research question? | 1.3. Are the conclusions adequately derived from the data? | 1.4. Is the interpretation of the results sufficiently supported by the data? | | 1.5. Is there consistency between qualitative data sources, collection, analysis and interpretation? | | | **Total (%)** | |  |
| **Abelkans et al. 2019** | Y | | | Y | | | Y | Y | Can’t tell | Can’t tell | | Can’t tell | | | 40% | |  |
| **Capranica et al. 2022** | Y | | | Y | | | Y | Y | Y | Y | | Y | | | 100% | |  |
| **Domingo et al. 2024** | Y | | | Y | | | Y | Y | Y | Y | | Y | | | 100% | |  |
| **Hallmann et al. 2019** | Y | | | Can’t tell | | | Can’t tell | Y | Y | Y | | Y | | | 80% | |  |
| **Henriksen et al. 2019** | Y | | | Y | | | Y | Y | Y | Y | | Y | | | 100% | |  |
| **Johnston et al. 2024** | Y | | | Y | | | Y | Y | Y | Y | | Y | | | 100% | |  |
| **Kerštajn et al. 2023** | Y | | | Can’t tell | | | Can’t tell | Y | Y | Y | | Y | | | 80% | |  |
| **Linnér et al. 2019** | Y | | | Y | | | Y | Y | Y | N | | Y | | | 80% | |  |
| **Nikander et al. 2020** | Y | | | Y | | | Y | Y | Y | Y | | Y | | | 100% | |  |
| **Nyberg et al. 2023** | Y | | | Can’t tell | | | Can’t tell | Y | Y | Y | | Y | | | 80% | |  |
| **Ryba et al. 2014** | Y | | | Can’t tell | | | Can’t tell | Y | Y | Y | | Y | | | 80% | |  |
| **Tekavc et al. 2015** | Y | | | Y | | | Y | Y | Y | Y | | Y | | | 100% | |  |
| **Thompson et al. 2022** | Y | | | Y | | | Y | Y | Y | Y | | Y | | | 100% | |  |
| **Reference** | | S1. Are the objectives and/ or research questions clear? | | | S2. Does the data collected address the research objective and/ or research question? | | 2.1. Is the sampling strategy appropriate for addressing the research objective and/or research question? | 2.2. Is the sample representative of the target population? | 2.3. Are the measures appropriate? | 2.4. Is the risk of non-response bias low? | | 2.5. Is the statistical analysis appropriate for answering the objective and/ or the research question? | | **Total (%)** | | | |
| **Abenza-Cano et al. 2020** | | Y | | | Y | | N | Y | Y | Can’t tell | | Y | | 60% | | | |
| **Conde et al. 2023** | | Y | | | Y | | Y | Y | Y | Y | | Y | | 100% | | | |
| **Condello et al. 2019** | | Y | | | Y | | Y | Y | Y | Y | | Y | | 100% | | | |
| **Fuchs and Wagner 2016** | | Y | | | Y | | Y | Y | Y | Can’t tell | | Y | | 80% | | | |
| **Gjaka et al. 2021** | | Y | | | Y | | N | Y | Y | Y | | Y | | 80% | | | |
| **Izzicupo et al. 2021** | | Y | | | Y | | N | Y | Y | Y | | Y | | 80% | | | |
| **Izzicupo et al. 2022** | | Y | | | Y | | Y | Y | Y | Y | | Y | | 100% | | | |
| **Maciá-Andreu et al. 2023** | | Y | | | Y | | Y | Y | Y | Y | | Y | | 100% | | | |
| **Mateo-Orcajada et al. 2022** | | Y | | | Y | | N | Y | Y | Can’t tell | | Y | | 60% | | | |
| **O'Neil et al. 2020** | | Y | | | Y | | N | Y | Y | Can’t tell | | Y | | 60% | | | |
| **Vaquero-Cristóbal et al. 2023** | | Y | | | Y | | Y | Y | Y | Y | | Y | | 100% | | | |
| **Reference** | S1. Are the objectives and/ or research questions clear? | | S2. Does the data collected address the research objective and/ or research question? | | | 3.1. Is there adequate justification for using a mixed methods design to address the research objective and/or research question? | | 3.2. Are the different components of the study effectively integrated in order to answer the research objective and/or research question? | 3.3. Are the results of the integration of qualitative and quantitative components adequately interpreted? | | 3.4. Are divergences and inconsistencies between quantitative and qualitative results adequately addressed? | | 3.5. Do the different components of the study adhere to the quality criteria of each tradition of the methods involved? | | | **Total (%)** | |
| **Rossi et al. 2021** | Y | | Y | | | Y | | Y | Y | | Y | | Can’t tell | | | 80% | |

% = percentage; Y = yes; N = no;
